# Feasibility of a Lifestyle Intervention Program for Prevention of Diabetes Among Women With Prior Gestational Diabetes Mellitus (LIVING Study) in South Asia: A Formative Research Study

**DOI:** 10.3389/fgwh.2020.587607

**Published:** 2020-11-27

**Authors:** Abha Tewari, Devarsetty Praveen, Pavitra Madhira, Lakshmi K. Josyula, Rohina Joshi, Suresh B. Kokku, Vandana Garg, Ishita Rawal, Kanika Chopra, Nantu Chakma, Sabrina Ahmed, Arunasalam Pathmeswaran, Pavithra Godamunne, A. S. Lata, Rakesh Sahay, Tulsi Patel, Yashdeep Gupta, Nikhil Tandon, Aliya Naheed, Anushka Patel, Deksha Kapoor

**Affiliations:** ^1^George Institute for Global Health, New Delhi, India; ^2^University of New South Wales, Sydney, NSW, Australia; ^3^Prasanna School of Public Health, Manipal Academy of Higher Education, Manipal, India; ^4^The George Institute for Global Health, University of New South Wales, Newtown, NSW, Australia; ^5^Manav Rachna International Institute of Research and Studies (MRIIRS), Faridabad, India; ^6^Centre for Chronic Disease Control, New Delhi, India; ^7^All India Institute of Medical Sciences, New Delhi, India; ^8^International Center for Diarrhoeal Disease Research Bangladesh (icddr,b), Dhaka, Bangladesh; ^9^Shiga University of Medical Science, Otsu, Japan; ^10^Faculty of Medicine, University of Kelaniya, Kelaniya, Sri Lanka; ^11^Sitaram Bhartia Institute of Science and Research, New Delhi, India; ^12^Department of Endocrinology, Osmania Medical College, Hyderabad, India; ^13^University of Delhi, New Delhi, India

**Keywords:** South Asia, gestational diabetes, lifestyle intervention, health worker, barriers and facilitators, prevention of type 2 diabetes mellitus

## Abstract

**Aim:** To refine and contextually adapt a postpartum lifestyle intervention for prevention of type 2 diabetes mellitus (T2DM) in women with prior gestational diabetes mellitus (GDM) in Bangladesh, India, and Sri Lanka.

**Materials and Methods:** In-depth interviews (IDIs) and focus group discussions (FGDs) were conducted with women with current diagnosis of GDM, and health care professionals involved in their management, to understand relevant local contextual factors for intervention optimization and implementation. This paper describes facilitators and barriers as well as feedback from participants on how to improve the proposed intervention. These factors were grouped and interpreted along the axes of the three main determinants of behavior–capability, opportunity, and motivation. IDIs and FGDs were digitally recorded, transcribed, and translated. Data-driven inductive thematic analysis was undertaken to identify and analyze patterns and themes.

**Results:** Two interrelated themes emerged from the IDIs and FGDs: (i) The lifestyle intervention was acceptable and considered to have the potential to improve the existing model of care for women with GDM; and (ii) Certain barriers such as reduced priority of self-care, and adverse societal influences postpartum need to be addressed for the improvement of GDM care. Based on the feedback, the intervention was optimized by including messages for family members in the content of the intervention, providing options for both text and voice messages as reminders, and finalizing the format of the intervention session delivery.

**Conclusion:** This study highlights the importance of contextual factors in influencing postpartum care and support for women diagnosed with GDM in three South Asian countries. It indicates that although provision of postpartum care is complex, a group lifestyle intervention program is highly acceptable to women with GDM, as well as to health care professionals, at urban hospitals.

## Introduction

Gestational diabetes mellitus (GDM) is defined as any degree of glucose intolerance first diagnosed in the second or third trimester of pregnancy and that is not clearly preexisting type 1 or type 2 diabetes (1). According to estimates from International Diabetes Federation (IDF), about one in every four pregnancies in IDF South East Asia region is affected by hyperglycemia, of which 90 percent is GDM. This compares with global estimates of one in every six pregnancies (2). In South Asia, the proportion of GDM ranges from 11% in Bangladesh (3), and 13.9% in Sri Lanka (4) to 25% in India (5–7), and is increasing, especially in developing countries (2). GDM is an established risk factor for future development of type 2 diabetes mellitus (T2DM), with high conversion rates within 5 years of childbirth, underscoring the need for urgent interventions to address risk factors and manage glucose metabolism in this population (8–12).

Interventions to manage and treat GDM emphasize adoption of a healthier lifestyle, with dietary planning and regular physical activity (13–15); and pharmacotherapy including oral agents such as metformin and insulin treatment, if necessary. Though earlier studies with lifestyle interventions in women with GDM, have shown a reduction in T2DM risk, these were mostly conducted in high- and upper-middle-income countries and with smaller sample sizes (16, 17), with sparse information on the use of low intensity lifestyle interventions in South Asian countries.

In the LIVING study (18), we are exploring the effectiveness of a resource- and culturally-appropriate lifestyle intervention provided to 1414 women with prior GDM, in Bangladesh, India, and Sri Lanka. Women with GDM, who do not have T2DM at 3–18 months postpartum, will be randomly assigned to intervention or usual care groups, and then followed up for a median of 18 months to determine the incidence of worsening glycaemic status. The intervention, delivered by minimally trained healthcare workers, comprises four group sessions focussed on healthy living, followed by two to four phone messages per week for 42 weeks, and additional individual coaching, if indicated by the non-achievement of participants' weight goals after the first 6 months of intervention.

The LIVING study was preceded by a formative study phase to refine and adapt the intervention for implementation in three countries. This paper describes the results of the formative phase that included exploration of postpartum care for women with GDM in public and private health facilities, and deliberations on optimal approaches to deliver a T2DM-prevention intervention (the LIVING intervention) using the existing health system's infrastructure and workforce, in Bangladesh, India, and Sri Lanka.

## Materials and Methods

### Study Design

In-depth interviews (IDIs) and focus group discussions (FGDs) were conducted to understand relevant local contextual factors, categorized as barriers and facilitators, for intervention optimization and implementation. These factors were grouped and interpreted along the axes of the three main determinants of behavior for the participants–capability, opportunity, and motivation. The study was undertaken from August 2016 to February 2017.

### Setting and Participants

A purposive sampling approach was used to select the study sites and participants (19). The study sites included one public and one private tertiary care hospital in each of North India, South India, and Bangladesh, and one public tertiary care hospital in Sri Lanka. All hospitals were located in urban areas and provided maternity care. The participants for IDIs included one hospital administrator, one obstetrician, one endocrinologist, and one non-physician health professional involved with obstetric or diabetes care (e.g., auxiliary nurse midwife, dietitian, or counselor), from each participating hospital. In Bangladesh, as there were no endocrinologists in the selected study sites, two endocrinologists from different public and private tertiary hospitals were invited to participate. Additionally, another obstetrician from a private hospital was interviewed in Bangladesh to gain a better understanding of local practice for the diagnosis and management of GDM. In addition, IDIs were conducted with women with a current diagnosis of GDM–three women in Bangladesh, seven in India, and one in Sri Lanka. Overall six FGDs were conducted; two in Bangladesh, three in India, and one in Sri Lanka. Postpartum women who were diagnosed with GDM in their most recent pregnancy, within the previous 24 months, participated in the FGDs. Women with a history of GDM were identified from the hospital records, and contacted, either by phone or during their visit to the hospital for routine care, for participation in this study.

### Data Collection and Analysis

All IDIs and FGDs were conducted in the local language of the participants (Bangla, Hindi, Sinhala, Telugu, or Tamil, based on the location) or in English, as per the convenience of the participants. The FGD guide and the IDI schedule, which included open-ended questions, were used to direct the discussions (Table 1). The IDIs and FGDs were conducted by a trained qualitative researcher with support from research team members. The interviews were conducted within the hospital premises, audio-recorded digitally, and transcribed verbatim in the local language. Each IDI took 35–40 min, and each FGD lasted around 60 min. Written informed consent was obtained from all participants for discussion and audio recording. Confidentiality was maintained throughout the study by removing the identifiers on audio recordings, interview notes, and transcripts.

**Table 1:** Focus group discussion and in-depth interview guide.

	**Interview questions and probes**
**IDIs with women with prior GDM**	Please tell us about yourself in brief. *Probes*: *Demographic details, family structure, resources, distance to hospital, etc*.
	**Current diagnosis and management of GDM**
	What do you know about Gestational Diabetes Mellitus? *Probe signs/symptoms/diagnosis*
	Please tell me about your experience of managing gestational diabetes? How was your experience with the hospital staff? *Probe*: *Support from family members, friends, relatives, any other, management strategies for GDM worked for you?* *What information do you have now, that would have been helpful at the beginning?*
	What information have you received from the hospital or the doctor? Do the discussions, with doctors involve postpartum care? *Probe*: *Healthy lifestyle postpartum, periodic testing, breastfeeding, contraception etc*.
	**Postpartum screening**
	Barriers and facilitators women may face in returning for an OGTT in the hospital in the postpartum period *Probe: Process of consultation; convenience; attitude/behavior of the staff*
	**Physical activity and healthy diet**
	How important is it to perform physical activity*/*exercise after childbirth according to you? How difficult*/*easy was it to find the time in your daily schedule?
	Have you done physical activity*/*exercise after the birth of your child? How much time did you spend on it? *Probe: Was it guided by your doctor/other support staff at hospital? What was the reaction/opinion of your family members? What type of support did you receive from family members?*
	What kind of activities do you think should be avoided after delivery?
	Nutritious food is required after childbirth. What type of food have you eaten after childbirth? *Probe: Was it suggested by the doctors/dietician at hospital/any other?*
	**Intervention**
	Do you think women with GDM need information for awareness about GDM? *Probe*: *Please elaborate and give reasons*
	What are the different ways that we can adopt to engage these women for our program? *Probe*: *Group sessions, individual sessions, SMS, phone calls, pamphlets, pictorial booklet, or any other*
	Explanation of intervention proposed and timelines
	Do you think such an intervention will be beneficial for women with GDM?
	Please suggest the best way you feel this intervention can be delivered. *Probe: At the hospital/mode of delivery/frequency of contact*
	Do you have any suggestions on how to keep women engaged in year 2 and 3?
	What are some ways to utilize the potential support from peers*/*family members to encourage sustainable behavior change?
	Explanation of content of sessions
	Who do you think will be the best person to deliver this intervention if this was to become a usual practice? Why? *Probe: doctor/nurse/counselor/MSW*
	What are the major challenges you expect during the delivery of the intervention? What may be some measures to overcome these?
	Do you feel this intervention should be a part of the usual care for GDM?
	How big should a group be? Why? *Logistics of group sessions*
	How long should one group session last?
	Which days do you think are good for group sessions? What time? Why?
	What is an appropriate place to conduct these sessions? Why this place? *Probe: Do you think we can take support from peers/family members of women? How can we engage their family members?*
	**Intrapregnancy session**
	Please provide feedback on the pamphlet. *Do you think this will be useful? What information can be included/excluded?*
**IDIs with Health Care Providers**	Please tell us about yourself. How long you have been associated with this institution? Probe: *Educational background, work experience, number of years and nature of work*
	**Current diagnosis and management of GDM**
	Please tell us about GDM. What are its sign and symptoms?
	What are the current practices in your hospital related to GDM? *Probe: Diagnosis and management practices, referral practices, and follow up*
	How many patients of GDM come to the unit, and how many get treated?
	In your opinion, what are the various factors that encouraged and discouraged individuals in seeking treatment*/*follow up? *Probe: Knowledge. support, socio-economic status, financial reasons, distance, taking care of children, any other*
	Lifestyle/behavior modifications are important for women with GDM. Please comment. *Probe*: *Barriers and challenges*
	Generally, what practices does this hospital follow to support these women for lifestyle*/*behavior changes?
	**Postpartum screening**
	What are the current practices related to postpartum screening of GDM cases? How is advice communicated and how much importance is it given during the interaction? *Probe: Advantages and disadvantages*
	According to you what are the potential barriers*/*facilitators in continuing the treatment? *Probe: Process of consultation, lab test, collection of results and re-consultation with doctor?*
	Do the women adhere to the visit schedule (postpartum OGTT)? Do they come back to see the doctor as advised?
	What should be our strategy if we plan to reengage with women after delivery? *Probe: Meeting them at the time of delivery, during hospitalization, contacting family, etc*.
	**Intervention**
	Do you think women with GDM in your hospital need some information to increase awareness, and improve their outlook toward GDM & its treatment? Probe: *Please elaborate and give reasons*.
	What are the different ways that we can adopt to engage these women for our program? *Probe: Group sessions, individual sessions, any other, group size, days, frequency, time*
	In your opinion who should be involved to provide such information with in the hospital? *Probe: ANMs, Doctors, Nurse, Counselor/MSW etc.? And who else?*
	According to you, do these people require some training? *Probe: Duration, content, frequency, place for such training*
	According to you, do we need to provide incentives to participants for their participation? If so, what type of incentives? Give details.
	How can we engage the peers*/*family members of women for this program?
	How can this program be sustained? Please suggest in what ways we can monitor program delivery? *Probe: Involvement of peers/family members of women, any other?*
	Overall, what are the major challenges you expect us to encounter while delivering the intervention program? Can you suggest measures to overcome these challenges?
**FGDs with women with prior GDM**	Please tell us about yourself in brief. At home, who takes the dietary decisions? *Probe: Demographic details, family structure, resources, distance to hospital, etc*.
	**Current diagnosis and management of GDM**
	What do you know about Gestational Diabetes Mellitus? *Probe: signs/symptoms/diagnosis*
	Please tell me about your experience of managing gestational diabetes? *Probe: Support from family members, friends, relatives, any other*
	Generally, what kind of difficulties did you face in managing your gestational diabetes? *-What management strategies (ways of dealing with your diabetes) worked for you?* *-What advice would you give to someone who was newly diagnosed with GDM?* *-What information do you have now that would have been helpful at the beginning?*
	Can you tell me a little about the information you received from the hospital or the doctor? Do the discussions with doctors involves postpartum care also? *Probe: maintenance of healthy lifestyle postpartum, periodic testing, breastfeeding, contraception, etc*.
	**Postpartum screening**
	What are various barriers and facilitators that women like you may face in returning for an OGTT in hospital in the postpartum period? *Probe: Process of consultation, lab test, collection of results and re-consultation with doctor, related costs, distance, convenience, time, mode of travel, attitude, and behavior of the staff*
	**Physical activity and healthy diet**
	How important is it to do physical activity*/*exercise after childbirth? Give details.
	Have you done physical activity*/*exercise after the birth of your child? How much time did you spend on it? *Probe:* *-Was it guided by your doctor/other support staff at hospital?* *-What was the reaction/opinion of your family members?* *-What type of support did you receive from family members?* *-How difficult/easy was it to find the time in your daily schedule?*
	In your opinion, what kind of physical activities*/*exercises should be avoided after delivery?
	Nutritious food is required after childbirth. Please give your opinion on this. Generally, what type of food have you been eating after childbirth? *Probe: Was it suggested by the doctors/dietician at hospital/family members/any other?* *Have you had any vitamins or iron supplements?*
	**Intervention**
	Do you think women with GDM in your hospital need some information to increase awareness, and improve their outlook toward GDM and its treatment? Probe: *Please elaborate and give reasons*.
	What are the different ways that we can adopt to engage these women in our program? Probe: *Group sessions, individual sessions, SMS, phone calls, pamphlets, pictorial booklet, or any other*
	Explanation of intervention proposed and timelines
	Do you think such an intervention will be beneficial for women with GDM?
	Please suggest the best way you feel this intervention can be delivered. Probe*: at the hospital/mode of delivery/frequency of contact*
	Do you have any suggestions on how to keep women engaged with us in year 2 and 3?
	What are the ways to utilize the potential support from peers*/*family members to encourage sustainable behavior change?
	Explanation of content of sessions
	Who do you think will be the best person to deliver this intervention if this was to become a usual practice? Why do you feel so? *Probe: doctor/nurse/counselor/MSW*
	What are the major challenges you expect women to encounter while this intervention is being delivered? Can you suggest measures to overcome these?
	Do you feel this intervention should be a part of the usual care for GDM?
	**Logistics of group sessions**
	How big should a group be? Why do you think this is an appropriate size?
	How much time should a group session last?
	Which days do you think are good for group sessions, and what time do you think will be the best to conduct group sessions? Why?
	What would be an appropriate place to conduct these sessions? Why do you suggest this place?
	Do you think we can take support from peers*/*family members of women? *Probe: how can we engage their family members*
	**Intra pregnancy session (Show pamphlet)**
	Please provide feedback on the pamphlet. Do you think this will be useful? What information can be included*/*excluded?

Study team members translated the transcripts into English (except in Bangladesh where Bangla transcripts were reviewed directly without translation) and cross-checked all translated transcripts with transcripts in the local language (also going back to the audio files whenever necessary) to ensure the quality of translation. Transcripts from India and Sri Lanka were then imported into QSR NVIVO (version 9.0) whereas the transcripts from Bangladesh were imported into Atlas.ti (version 7.5) qualitative data analysis software for structuring and analysis of the data (20). Data-driven inductive thematic analysis (21, 22) was undertaken to identify and analyze patterns and themes. Any emerging subthemes were identified during analyses and summarized separately. Each theme was conceptualized as an influencing “factor” on the management of GDM and lifestyle related decisions. We adhered to the COREQ guidelines for reporting qualitative research (23).

**Figure 1:**
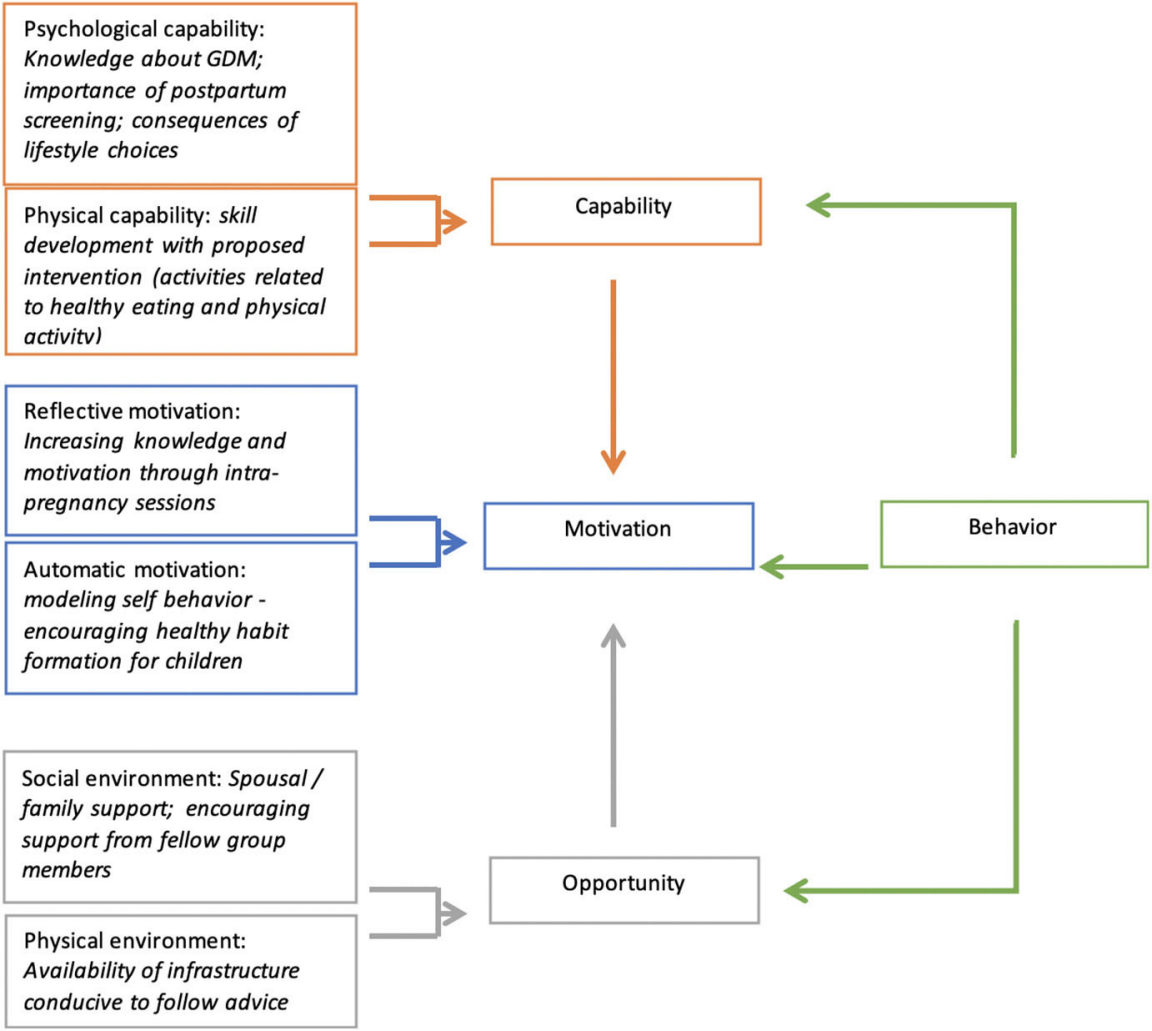
Applying the constructs of the COM-B behavior change framework to the themes generated in the analysis.

The themes generated in this analysis for all participants were further mapped to the constructs of the COM-B model, developed by Michie et al. (24), which proposes that behavior (B) is the result of interaction among capability (C), opportunity (O), and motivation (M). The COM-B model suggests that for a person to engage in a specific behavior, (s)he needs to: (i) be psychologically and physically able; (ii) have the physical and social opportunity; and (iii) want or need to do the change in behavior. The model is illustrated in Figure 1. Along with the information on postpartum care to optimize the postpartum T2DM prevention intervention, information was also collected to understand the awareness among women about GDM and how the disease had postpartum implications. This helped us understand the relevance of GDM care on their acceptability of continued care after childbirth for preventing future metabolic complications.

### Ethics Approval

Ethics approval was obtained from the Human Research Ethics Committees of the All India Institute of Medical Sciences, Delhi, India (Ethics number IEC/NP-294/05.09.2014); University of Sydney, New South Wales, Australia (Ethics number HE 2016_177); University of Kelaniya, Sri Lanka (Ethics number P/163/10/2016); and International Center for Diarrhoeal Disease Research, Bangladesh (icddr,b), Bangladesh (Ethics number PR 17012).

## Results

### Characteristics of Participants

Overall, 41 IDIs and 6 FGDs were conducted in the three countries. The IDIs included interviews with health care providers (HCPs) (endocrinologist, obstetrician, healthcare administrator, gynecologist, nurse, counselor, and health worker) and postpartum women diagnosed with GDM in the previous 24 months. The FGDs were conducted only in postpartum women diagnosed with GDM in the previous 24 months.

Participants were from diverse socio-demographic backgrounds, and had varying levels of literacy and employment. In Bangladesh, most women lived in joint families, whereas in India and Sri Lanka, participants' residential situations were evenly divided between nuclear and joint families. Details of the study sites and participants are presented in Table 2.

**Table 2:** Characteristics of participants in FGDs and IDIs in three countries.

**Variables**	**Bangladesh**	**India**	**Sri Lanka**
Sites	2 Hospitals (1 public and 1 private)	4 Hospitals (2 public, 2 private) from one Northern and one Southern state	1 Public Hospital
**FGDs WITH WOMEN DIAGNOSED WITH GDM**			
FGDs and number of participants	2 FGDs (*n* = 13)	3 FGDs (*n* = 18)	1 FGD (*n* = 6)
Participants' age in years (mean and range)	26.6 (18–34)	27 (18–36)	30.5 (26–35)
**Education**			
No education	–		
Primary	1		
Secondary	4	2	
Intermediate and above	8	16	6
**Occupation**			
Homemaker	10	11	5
Student	1	–	
Entrepreneur/business	–	2	
Professional	2	5	1
**Type of family**			
Nuclear family	3	8	1
Joint family	10	10	5
Distance from the nearest hospital			
<5 kms	8	18	5
>5 kms	5		1
**IDIs WITH HEALTH CARE PROVIDERS**			
Number of participants	9	17	4
Participants' age in years (mean and range)	51.2 (40–58)	40 (25–55)	45.5 (32–59)
**Gender**			
Male	3	6	2
Female	6	11	2
Education			
Intermediate and above	9	17	4
**Area of expertise**			
Hospital administrator	2	4	1
Gynecologist	3	2	0
Obstetrician	–	4	1
Endocrinologist	2	5	1
Nurse	–	1	0
Non-physician health worker (including counselor)	2	1	1
**IDIs WITH WOMEN DIAGNOSED WITH GDM**			
Number of participants	3	7	1
Participants' age in years (mean and range)	26.6 (18–34)	30.5 (25–36)	32
Education	Not available		
No education		–	
Primary		1	
Secondary		2	
Intermediate and above		4	1
**Occupation**			
Homemaker	3	5	1
Student			
Entrepreneur/business			
Professional		2	
Type of family	Not available		
Nuclear family		5	1
Joint family		2	–
**Distance from the nearest hospital**			
<5 kms	3	7	1
5–7 kms	–		

We present the summarized pooled findings from three countries in two sections—(i) facilitators and barriers for improvement of usual GDM care; and (ii) feedback for optimization of the intervention. In situations where the discussions generated findings specific to each country, they are presented under the respective thematic category, and the country is identified. Table 3 presents the summary of the themes and key findings of the study mapped to the constructs of COM-B and domains identified in the behavioral analysis.

**Table 3:** COM-B components linked with key findings of LIVING formative study.

**COM-B component identified in the behavioral analysis**	**Domains linking to COM-B component**	**Themes**	**Key findings**
Capability (Psychological and Physical)	Knowledge	Knowledge of GDM	- Women had basic or limited knowledge of GDM, its treatment and management. - Most of them became aware of GDM after being diagnosed. - HCPs observed a lack of knowledge among women about GDM.
	Skills	Current practices and criteria for treatment	- HCPs followed different guidelines for diagnosis and treatment of GDM. These included mainly three-point OGTT, NICE and American guidelines. - Information to maintain healthy lifestyle was provided by HCPs - HCPs informed these women about importance of physical activity, regular follow-ups, family planning & breast-feeding.
Opportunity	Social (societal influences)	Challenges related to management of GDM	- Family role perceived as both supportive and hindrance in following the routine after childbirth. - Long waiting time at the hospital - Women accessing the government facilities expressed difficulty in standing in a queue for long hours. - Lab test dates being given too far in the future.
	Physical (environmental)	Challenges to maintain healthy lifestyle	- Lack of time to cook, low quality of available food items, easy access to cooked food, and peer pressure to eat unhealthy food were barriers to maintain healthy lifestyle. - Family-related factors, such as prioritizing others in the family over oneself, were reported barrier to take care of oneself. - Limited financial resources deterred them to prepare the wholesome meal.
Motivation	Belief about capabilities and consequence		- Priorities change after childbirth. Primary motivation for the lifestyle modification during pregnancy was the health of unborn child
	Emotion		- Majority of women reported being shocked and experiencing fear and anxiety after diagnosed with GDM. - HCPs shared that most of the patients get panicked after diagnosis with GDM.

#### Facilitators and Barriers for Improvement of Usual GDM Care

##### Improving Knowledge for Care

Although women with GDM revealed that they were aware of the condition, the level of information about the causes of GDM, and its attributes, varied. In India and Sri Lanka, a few women attributed GDM to diet and lifestyle, whereas some others attributed it to weather conditions. Healthcare providers (HCPs) were the main source of information in all three countries. In Bangladesh, relatives and friends with GDM were cited as an additional source of information on GDM. HCPs, especially clinicians, noted a lack of understanding and knowledge related to GDM among women, particularly in those with low levels of literacy, and from families with little to no formal education.

“*…I know about diabetes, but I didn't know that it happens in the pregnancy period.” (FGD, patient, Bangladesh)*

“*Most of the patients don't know exactly what is the GDM and why it has come... until and unless we explain them.” (IDI, HCP, India)*

Almost all participants reported that women with GDM felt fear and anxiety on being diagnosed with GDM. A few HCPs opined that information of increased risk of developing T2DM in the future would motivate women to participate in a lifestyle intervention program. Most of the HCPs felt that there was a great need for special counseling sessions to be held for women with GDM, and, in addition, the staff and the facilitators also needed to be provided with refresher courses for lifestyle modification and medical management of GDM. Counselors did not consider themselves diabetes experts.

“*...We are not diabetes specialists. We just learnt about GDM by reading materials. If we have to counsel the patient then we need to learn more about diabetes and gestational diabetes. We need to know about the behavioral modification like diet, physical activity etc. If we can learn these things we will be able to provide it to the patients easily.” (IDI, Counselor, Bangladesh)*

In all the study sites, HCPs spoke about providing information on maintaining a healthy lifestyle, emphasizing healthy food, regular follow-up, family planning, and breastfeeding. However, women with GDM reported that their HCPs advised them almost exclusively on healthy diet. Women in India and Sri Lanka reported some mention of physical activity and regular monitoring of blood sugar, whereas women in Bangladesh reported not getting any suggestion about physical activity in the period immediately following childbirth.

“*Should take nutritious food, it is important to take foods that are suitable for diabetics like that Thebu leaves and red rice” (FGD, patient, Sri Lanka)*

“*Yeah, we counsel them and then we refer them to the dietitian. They tell the patient about total calorie requirement and how she has to divide the meals and then we tell them to take the medical nutrition therapy and check blood sugar profile after maybe two weeks to see the improvement.” (IDI, HCP, India)*

##### Improvement of Routine Care for GDM After Childbirth

HCPs from all the three countries noted that continuous follow-up of women with GDM after the 6 weeks postpartum consultation is not routine practice. The follow-up care and counseling sessions for women post childbirth, in the proposed intervention, were welcomed by them.

“*Only opportunity we have is six weeks postpartum, but after that usually if they have diabetes, they are being referred to the physician or the endocrinologist for follow up. We don't have a continuous follow up with them.” (IDI, HCP, Sri Lanka)*

A major component of the proposed intervention is the placement of an auxiliary nurse midwife (or equivalent) in charge of the counseling sessions postpartum. This was very positively received, given the several challenges cited by the patients related to the long waiting time for doctor consultations, or any laboratory investigation. Most participants admitted to finding it difficult to leave their babies in somebody else's care, during their postpartum hospital visit. Though many HCPs welcomed this approach, HCP from Sri Lanka opined that all the service providers of the hospitals should be included so that anyone could provide the care in the absence of others.

“*I think counselor would be able to do it. The messages (in the intervention) are general and not too difficult. Counselor is already delivering health messages to the patients and you need to just add your messages. I think counselor is enough for this.” (IDI, Gynecologist, Bangladesh)*

“*I think even the nursing officer, the nutritionist… now it has to be in a way, if it can be organized as a teamwork, it will be better. With these nursing officers, nutritionist and VOG [Obstetrician and Gynecologist] and all, we can have a combined team effort.” (IDI, hospital administrator, Sri Lanka)*

##### Social Support

Many FGD and IDI participants considered their husband or other family members, mostly their mother or mother-in-law, as their primary source of support, reminding them to take their medicines, accompanying them to hospitals, and sometimes helping in cooking healthy meals. Most also acknowledged the support, including psychological support in some cases, that they got from health professionals, including doctors, dietitians, diabetes educators, and the primary care team; however, the level of this support varied among the participants depending upon the hospitals that they went to for treatment. Neighbors were also mentioned as a source of support in Sri Lanka, specifically neighbors cooking special food (diabetic food) for women with GDM.

“*I didn't have any problem, rather, I had lot of cooperation from my family. My husband, mother-in-law, everyone helped me by suggesting when and which food to eat and which not to eat. Sometimes they also avoided some foods which were forbidden for me.” (FGD, patient, Bangladesh)*

In India and Bangladesh, a few women reported that family members encouraged them to engage in some physical activity when their babies were asleep, but mentioned that they needed to do so indoors so as to be within easy reach of their babies.

##### Reduced Priority of Self-Care

In India, many women with GDM stated that the health of the unborn baby before childbirth, and that of the newborn after childbirth, were of high priority for them. This acted as a primary motivator for healthy lifestyle modification during pregnancy, but after childbirth, childcare took a lot of their time and energy, leaving them less time and effort to devote to themselves. HCPs were also of the same opinion and favored the planning of the intervention around the child's hospital visits for routine immunization. One of the endocrinologists from Bangladesh suggested motivating women with GDM during their pregnancy to undergo postpartum oral glucose tolerance testing (OGTT). In addition, he considered proper counseling of the family members, such as the husband or mother-in-law, very important.

“*….Then for the next 9 months I strictly followed the advises given by them [doctors] on diet modifications just for the health of baby.” (FGD, patient, Sri Lanka)*

“*It is not easy to take out time for own self…* (laughing) *to take care…. Most of the time is spent with the baby.” (IDI, patient, India)*

##### Household Conditions and Societal Influences

In India, most of the FGD participants opined that it was difficult to follow a schedule and diet plan with extended families around them. Childbirth, a happy occasion, often involved protracted visits from the participants' parents and parents-in-law. In such situations, as meals were prepared in a single kitchen, it was difficult to adhere to separate menus, or alter the entire family's meals to suit the new mother's dietary requirements. Strongly positive or negative beliefs in the family around certain foods made it difficult for women to follow the diet plan drawn up by their HCPs. They felt that too much emphasis on a special diet ultimately led to disruptions and tensions in the family. On the other hand, women from nuclear families highlighted their lack of time to cook proper food, the low quality of available food products, and easy access to unhealthy cooked food or junk food, as reasons for their inability to follow a healthy diet plan.

“*Doctors suggest not to eat ghee as well but again the dilemma is I am in a condition that I don't know if what the doctor gives me will give me more strength or what my mother-in-law gives me will give me more strength.” (IDI, patient, India)*

“*Family support is important but at times, food choice of my husband and family members is different... so I need to cook food as per their choice.” (FGD, patient, Sri Lanka)*

Though most women understood the importance of physical activity postpartum to reduce the risk of developing type 2 diabetes, constraints of weather conditions, time, and place were cited as reasons for the inability to exercise.

“*The thing is my son is one year old. The moment I think to go for a walk, I have to prepare the food for him… everything has to be ready. Because the caretaker cannot do anything during the day. She has to be with the baby, because, he is crawling, he's trying to walk, he's standing… so much mess.” (FGD, patient, India)*

In addition, in Bangladesh, security issues such as risk of getting kidnapped were reported by the FGD participants as a barrier to walking outside. One of the FGD participants said,

“*….Some do not open the gate of their house till 7am and it may be problematic. The main problem is kidnapping in the city.” (FGD, patient, Bangladesh)*

#### Feedback for Optimizing the Intervention

##### Content of the Intervention

Many women with GDM felt the need to receive detailed information on GDM and its management, along with information related to breastfeeding, and other baby-focused issues, especially in India. They also expressed a requirement for family members, especially husbands and mothers-in-law, to be provided this information.

“*Many women know about it (GDM) from six months gestation and they get service for next three months. At that time we had to visit another hospital (by referral) because there is no service about diabetes in this hospital which is essential for us.” (FGD, patient, Public hospital, Bangladesh)*

HCPs in all the three countries reiterated the importance of creating awareness about GDM and maintaining a healthy lifestyle. They suggested the inclusion of training content about the importance of insulin, adherence to medications, healthy eating habits, physical activity, and regular follow-up visits with the doctor. HCPs from India thought that yoga and meditation could be helpful strategies to maintain a healthy regimen and suggested their inclusion in the information booklet.

The counselors in Bangladesh shared that they need training in GDM and its management prior to counseling patients. In particular, they wanted more information about medications and use of insulin. A few endocrinologists mentioned that information such as the cut off ranges for the diagnostic criteria for GDM needs to be imparted to obstetricians as well. One of the counselors in Bangladesh emphasized psychological counseling for women who had GDM, as diabetes patients could get worried, and even become depressed.

“*In addition to diet, regular checkup, physical activity, regular medication the patients need to be mentally stable. Because sometime mental stress can cause diabetes. Mental comfort means to give courage and support to the patients. Patients usually get afraid when they get diabetes. That time patients need to be supported mentally so that they can feel less stress.”* (IDI, Counselor, Bangladesh)

##### Mode of Intervention Delivery

*Group Sessions vs. Individual Counseling Sessions*. All participants, including HCPs, preferred group counseling sessions with fewer than 10 persons in a group for intervention delivery owing to ease of delivery of intervention, peer influence for effective learning, and the saving of time for the HCPs. Group sessions were considered to offer an opportunity to know others with similar challenges, address common issues, give and receive support, and encourage interpersonal interaction.

“*If you can arrange group session, it will be much effective than others…. If a number of people sit together it will more effective because they can share with one another.” (IDI, HCP, Bangladesh)*

However, one gynecologist from a private hospital in Bangladesh mentioned difficulty in arranging groups as she thought housewives are very busy and allocating time for the group sessions could be challenging for new mothers. She suggested alternatives like mass media, e.g., television, radio, and social media for intervention delivery.

*Accompanying patients to sessions*. IDIs and FGDs with women with GDM highlighted that most of them wanted other family members, especially husbands and mothers-in-law, to accompany them to the sessions, as that would ensure that there was someone to care for their babies while they attended the sessions, and also help their family members realize the importance of the messages conveyed in the sessions, and, thereby, prevent arguments and opposition back at home.

“*If you will not tell the family, in most of the families, the patient will not come. The mother-in-law will not allow her to leave the baby at home and then go to the hospital for just her own counseling… So I think husband and mother-in-law should be told and counseled about the importance.” (IDI, HCP, India)*

*Duration of session*. The suggested duration for each session varied from 40 min to 2 h. The majority of the participants were in favor of 30 to 60-min sessions, whereas some women with GDM, specifically in India, felt that more time, up to 2 h, might be required. Almost all the participants in India and Sri Lanka preferred sessions during weekends, especially during the morning or at noon, as babies were most likely to be asleep during those times.

*Session delivery*. Almost all women with GDM preferred to receive the intervention (counseling sessions) from doctors or clinicians with a high level of expertise on the topic. However, in view of the time constraints experienced by doctors, trained personnel, such as nurses, diabetes educators, and nutritionists, were also seen to be fit for this role. The doctors, due to paucity of time, preferred the trained health workers to implement the sessions, under their guidance. A few participants wanted the intervention to be delivered at their homes rather than at hospitals, and, for this purpose, they felt that non-physician health workers, such as community health workers, and community health volunteers, e.g., accredited social health activists (ASHAs) in India, would be the best to deliver the intervention.

“*ANM… yes, and trained social worker also. The person should be aware of the disease, its complications and lifestyle modifications related to it… basically this person should be knowledgeable.” (IDI, HCP, India)*

“*A medical officer can be useful as the consultants won't be able to devote proper time. Counselor can be used to support the consultant however; the counselor can't understand the technical issues. Some of our medical officers also provide counseling services.” (IDI, hospital administrator, Bangladesh)*

*Additional Reading Materials for Use at Home*. Along with group counseling sessions, many women with GDM wanted additional materials, e.g., pictorial pamphlets, to take home. They felt that such materials would help them communicate health messages of relevance to other members of their family. They observed that pamphlets with textual information might not be of much use for illiterate participants.

*Text messages vs. voice calls*. Text messages were preferred by most participants over pre-recorded voice messages sent as voice calls, however, they noted that it would be difficult for women who were illiterate to benefit from text messages. Alternatively, some participants including HCPs favored the idea of making telephonic calls for reminders.

“*It seems that, if you send any message I have to read it. But there are many women who can't read messages from the mobile phone, like me. I have studied very little, I can't read it but my husband can. There may be many women who can't read SMS. So, it is better if you send a call.” (FGD, patient, Bangladesh)*

*Number of sessions*. A few respondents preferred only one face-to-face session, with booster text messages instead of further sessions as long as the prescribed regimen were followed, and blood sugar maintained. Tapering off of sessions based on desired levels of progress was also suggested by some participants.

“*I think it should be every month in the beginning. Maybe in the first four to six months it should be every month and then we have to select those people who are doing it, who are motivated. Those who are understanding well, for them we can reduce the frequency. We can give them SMSs if you are a person with good engagement scheme. So that is what we have planned in our intervention.” (IDI, HCP, India)*

*Venue for imparting sessions*. Except for a few women who wanted the intervention to be delivered at their homes, most preferred the nearby hospital (neighborhood hospital or Ministry of Health (MoH) office in Sri Lanka; primary health care center or government hospitals in Bangladesh and India) as the site for group sessions. They felt that it would help them interact with other participants as well as allow them to access the laboratory facilities, if required. A few HCPs pointed out an insufficiency of space in the hospitals for conducting group sessions. Consultation or waiting rooms in India and Sri Lanka, and counseling rooms in Bangladesh were a few alternatives identified, by HCPs, as venues for group sessions. They also suggested that the hospitals ensure supportive facilities for childcare during the sessions.

*Recruitment and First contact with women with GDM*. For recruitment into the intervention, HCPs in all three countries felt that women with GDM should be approached during pregnancy and encouraged to undertake the intervention postpartum. With blood sugar levels normalizing after childbirth among women with GDM, the HCPs expected them to be likely to feel that they have recovered and will not need any more attention post-delivery. The HCPs opined that early contact and keeping in touch thereafter would enhance the likelihood of participants' attending the intervention group sessions regularly.

“*Best time I would like to add would be before delivery. Because prior to that they are very much concerned about the outcome of their pregnancy. (IDI, HCP, India)*

Some hospital administrators suggested that combining the intervention visits with other routine hospital visits (for e.g., family planning procedures and immunization of baby) would reduce the burden on the mothers to make special trips to the hospital. The best time for reengagement with the patient after childbirth was judged to be 6–8 weeks postpartum, when immunization of the child would begin. Most HCPs emphasized that involving other departments, if available, including gynecology, endocrinology, pediatrics, and laboratory, in the intervention would lead to proper implementation of the intervention, and engage women with GDM. They felt that involving community health workers could help the roll-out as well as the scale-up of the intervention.

“*At the national level, majority of delivery takes place in community district hospitals where you have health workers already going to the community, so they can help. As such they are making post-natal visits and we can ask them to do additional visits.” (IDI, HCP, India)*

Providing frequent reminders, keeping the appointment dates flexible, and incentivizing women for participation through travel reimbursement were considered useful strategies to maximize participation in the intervention by a few HCPs in India and Bangladesh.

“*Sometime it is not possible to provide incentives. But, at least if there is any system like food arrangement or provision of travel cost, then the patients would get interest.” (IDI, HCP, Bangladesh)*

## Discussion

This qualitative study sought to understand the factors influencing postpartum care and support for women diagnosed with GDM, in Bangladesh, India, and Sri Lanka. This study also explored the possible barriers and facilitators, through the COM-B model, of implementing a lifestyle intervention program administered after childbirth, for the prevention of future T2DM among women diagnosed with GDM.

Perceived barriers were mainly related to individual and contextual factors. At the individual level, similar to the findings from a review examining women's experiences of a diagnosis of GDM among 34 studies (25), most women in this study had basic or limited knowledge of GDM, and its treatment and management. This influenced the initial reaction to the diagnosis which was mostly fear and anxiety, highlighting the need to enhance awareness of GDM, and associated risk factors and consequences (26, 27). This is essential at diagnosis, or even earlier in the pregnancy, as routine antenatal care information (28). In the postpartum period when women come to the hospital for healthcare for their children, they can be provided with information on the importance of physical activity, healthy diet, and regular follow-up after GDM (26). The proposed intervention strategy could increase opportunities for women to access group-based intervention at the hospital postpartum.

Our findings highlighted a perceived need to coordinate care between obstetricians (driving pregnancy care) with primary care physicians, endocrinologists, and diabetologists (driving postpartum care). Other researchers have pointed to the importance of integration of various departments in hospitals for the treatment and management of various diseases (29), especially formalized obstetrician-primary care provider hand-offs for women with GDM (30). In line with several other studies, this study found that the postpartum follow-up of women for blood glucose testing was considered the main challenge in access to quality care (31–33). This situation is unlikely to improve unless proper guidelines to treat GDM, and to follow up patients after childbirth are outlined.

Most women acknowledged the support that they received from family members, especially husbands and mothers-in-law, to maintain a healthy lifestyle during and after pregnancy. Some studies have indicated the potential for long-term positive impact of such support, which can be an agent of continuous motivation to follow a healthy lifestyle during and after pregnancy (34, 35). On the other hand, extended families and childcare take up a large proportion of new mothers' time and energy leading to low priority of self-care. Findings from this study underscore previous research that indicates the importance of enhancing individuals' knowledge, and also of creating supportive environments to facilitate the adoption of a healthy lifestyle during and after pregnancy (35, 36).

The proposed intervention aims to provide the required information in a setting convenient for participants, as well as opportunities for discussion with peers on the best way to use such information. Inviting the patients' family members to group counseling sessions is a way to enhance interest, impart awareness, and promote social support among families and peers (37, 38). Besides information sessions, participants requested additional pictorial materials to take home to communicate health messages to other family members, consistent with other studies that highlight the value of using pictorial material to sensitize people (39).

The proposed postpartum lifestyle intervention was acceptable to all women with GDM and the HCPs, and suggestions were provided to refine it, and tailor it to the local contexts. The present study findings similar to findings from other studies, suggest a strong need for a lifestyle-based intervention program suitable to women accessing both government and private hospitals and delivered at the hospital as per their convenience, ideally at 6–8 months postpartum (27, 38).

Regular phone calls were suggested as an effective way to keep in touch with participants and promote adherence to the program, in agreement with findings from other studies showing improved adherence and glycaemic control in women in regular telephonic contact with interventionists (40, 41). The participants in this study, especially those from Bangladesh, highlighted the potential usefulness of providing travel reimbursement to the women during the intervention, supporting the observation that the use of incentives in health services may promote long-term sustainability of an intervention (42).

### Strengths and Limitations

The major strength of this study is its representation of the overall situation in urban areas in South Asia, as it collects data from three countries in South Asia, representing a large population size. The principal limitation of this study is that it may not represent the rural perspective, as it was conducted in urban settings thereby limiting the generalization of the findings to rural settings. The other limitation is that the education status of the participants was not uniformly elicited thereby limiting the correlation of the findings with the educational level of the participants. All the participating women in India and Sri Lanka were literate (minimum of primary and maximum of intermediate and above), while this information is not available for participants in Bangladesh.

## Conclusion

This study indicates that though provision of postpartum care is complex, the proposed strategy is acceptable to women with GDM, as well as to HCPs involved in treating them, at urban hospitals. The findings also inform the refinement of a group intervention for prevention and management of T2DM among women with GDM. This intervention will be tested in a randomized controlled trial to determine its effectiveness and cost-effectiveness in improving GDM related care in three South Asian countries, viz., Bangladesh, India, and Sri Lanka.

## Data Availability Statement

The data set from this study are held securely at the George Institute. Researchers who wish to use data are encouraged to write to the George Institute Data Access Coordinator who can be contacted at datasharing@georgeinstitute.org. Access to datasets will be facilitated to those who meet pre-specified criteria for confidential access, available at https://www.georgeinstitute.org/data-sharing-policy.

## Ethics Statement

The studies involving human participants were reviewed and approved by Human Research Ethics Committees of the All India Institute of Medical Sciences, Delhi, India (Ethics number IEC/NP-294/05.09.2014); University of Sydney, New South Wales, Australia (Ethics number HE 2016_177); University of Kelaniya, Sri Lanka (Ethics number P/163/10/2016); and International Center for Diarrhoeal Disease Research, Bangladesh (icddr,b), Bangladesh (Ethics number PR 17012). The patients/participants provided their written informed consent to participate in this study.

## Author Contributions

AT, DP, LJ, and DK: had full access to all the data in the study and take responsibility for the integrity of the data and the accuracy of the data analysis. APate, AN, NT, YG, RJ, AT, TP, DP, and DK: concept and design. AT, DP, PM, LJ, RJ, SK, VG, IR, KC, NC, SA, APath, PG, AL, RS, YG, NT, AN, APate, and DK: acquisition, analysis, or interpretation of data. AT, DP, PM, LJ, and DK: drafting of the manuscript. AT, DP, DK, AN, NC, and SA: analysis. APate and NT: obtained funding. AT, PM, LJ, RJ, SK, VG, IR, KC, NC, SA, PG, AL, RS, YG, and DK: administrative, technical, or material support. APate, NT, DP, AN, and APath: supervision. All: critical revision of the manuscript for important intellectual content.

## Conflict of Interest

The authors declare that the research was conducted in the absence of any commercial or financial relationships that could be construed as a potential conflict of interest.
